# Thrombelastometry guided blood-component therapy after cardiac surgery: a randomized study

**DOI:** 10.1186/s12871-019-0875-7

**Published:** 2019-11-06

**Authors:** Martin Haensig, Joerg Kempfert, Pia-Maria Kempfert, Evaldas Girdauskas, Michael Andrew Borger, Sven Lehmann

**Affiliations:** 1 Department of Vascular Surgery, Cardiovascular Center, University of Leipzig, Liebigstr 20, 04103 Leipzig, Germany; 2 Department of Cardiothoracic and Vascular Surgery, German Heart Center Berlin, Berlin, Germany; 3 Department of Cardiac and Cardiovascular Surgery, University Heart Center Hamburg, Hamburg, Germany; 4 Clinic of Cardiac Surgery, Heart Center, University of Leipzig, Leipzig, Germany

**Keywords:** Cardiac surgery, Bleeding, Point-of-care testing, Thrombelastometry, Blood-component therapy, Transfusion

## Abstract

**Background:**

Significant bleeding is a well known complication after cardiac surgical procedures and is associated with worse outcome. Thrombelastometry (ROTEM®) allows point-of-care testing of the coagulation status but only limited data is available yet. The aim was to evaluate the ROTEM®-guided blood component therapy in a randomized trial.

**Methods:**

In case of significant postoperative bleeding (> 200 ml/h) following elective isolated or combined cardiac surgical procedures (including 14% re-do procedures and 4% requiring circulatory arrest) patients were randomized to either a 4-chamber ROTEM®-guided blood-component transfusion protocol or received treatment guided by an algorithm based on standard coagulation testing (control). One hundred four patients (mean age: 67.2 ± 10.4 years, mean log. EuroSCORE 7.0 ± 8.8%) met the inclusion criteria. Mean CPB-time was 112.1 ± 55.1 min., mean cross-clamp time 72.5 ± 39.9 min.

**Results:**

Baseline demographics were comparable in both groups. Overall there was no significant difference in transfusion requirements regarding red blood cells, platelets, plasma, fibrinogen or pooled factors and the re-thoracotomy rate was comparable (ROTEM®: 29% vs. control: 25%). However, there was a trend towards less 24-h drainage loss visible in the ROTEM®-group (ROTEM®: 1599.1 ± 834.3 ml vs. control: 1867.4 ± 827.4 ml; *p* = 0.066). In the subgroup of patients with long CPB-times (> 115 min.; *n* = 55) known to exhibit an increased risk for diffuse coagulopathy ROTEM®-guided treatment resulted in a significantly lower 24-h drainage loss (ROTEM®: 1538.2 ± 806.4 ml vs. control: 2056.8 ± 974.5 ml; *p* = 0.032) and reduced 5-year mortality (ROTEM®: 0% vs. control: 15%; *p* = 0.03).

**Conclusion:**

In case of postoperative bleeding following cardiac surgical procedures a treatment algorithm based on “point-of-care” 4-chamber ROTEM® seems to be at least as effective as standard therapy. In patients with long CPB-times ROTEM®-guided treatment may result in less bleeding, a marked reduction in costs and long-term mortality.

**Trial registration:**

German Clinical Trials Register, TRN: DRKS00017367, date of registration: 05.06.2019, ‘retrospectively registered’.

## Background

Postoperative impairment of hemostasis is a well known phenomenon associated with cardiac surgical procedures due to the operative trauma, cardiopulmonary bypass, heparinisation and subsequent reversal and other factors [1, 2]. Significant postoperative bleeding has to be expected in 5–10% of patients [3, 4] and is associated with significantly worse outcome and an up to 8-fold increase in mortality [4]. Although it has been estimated that cardiac surgery accounts for 20% of all national blood products consumption in the United States [3] strategies for blood component therapy demonstrate a great variability [5]. Given the known side-effects of blood components and costs it is of concern that a substantial proportion of these products seems to be transfused unnecessarily [6]. Or in other words: the surgical center has been identified as an independent risk factor for transfusion [5].

Well-structured blood component therapy protocols based on standard coagulation testing have been proven to be beneficial in comparison to commonly used “empiric” treatment [7]. Treatment algorithm guided by “point-of-care” thrombelastometry (ROTEM®) might be associated with further benefits as they are capable to specifically identify potential coagulation disorders in a timely manner allowing specific and fast treatment [8, 9]. However, for cardiac surgical patients only limited data is available from a few studies [10, 11].

The aim was to assess efficacy of a ROTEM®-guided blood component treatment algorithm for elective cardiac surgical patients in comparison to a protocol based on standard coagulation testing in a prospective randomized trial.

## Methods

### Study design

After approval by the local ethics committee of the University of Leipzig (reference nr.: 049/07-ek), and written consent obtained from each patient, 104 patients scheduled for elective cardiac surgical procedures were enrolled in this single center study at the Heartcenter, Leipzig. Patients that demonstrated significant persistent postoperative bleeding defined as drainage loss of 100 ml / 30 min or more than 200 ml / h after arrival at the intensive care unit (ICU) were randomized. The first group (ROTEM®) were treated based on a thrombelastometry-guided (4-chamber ROTEM™) blood-component transfusion protocol whereas the other group (Control) received treatment guided by a protocol based on standard coagulation testing.

Primary endpoints were transfusion requirements regarding red blood cells, platelets, plasma, fibrinogen or pooled factors. The secondary endpoints were 24-h drainage loss, re-thoracotomy rate and cost analysis of blood and coagulation products.

During the study period of 2^1^/_2_ years, a total of 6041 patients underwent elective cardiac surgical procedures and the rethoracotomy rate was 8.3% at our center.

### Subgroup analysis of patients at “high-risk” for bleeding (long CPB times)

Since patients with long CPB-times are known to exhibit an increased risk for diffuse coagulopathy, a priori a subgroup analysis regarding this specific risk factor was planned. Regarding the recent literature, an appropriate definition of the term “prolonged CPB time” is unclear [12, 13]. Nevertheless, we choose the mark of 115 min, because this was the mean of our CPB times based on our preliminary study results. However, and most importantly post hoc all CPB times ≥115 min revealed evidence for a significant difference.

### Patients

Preoperative antiplatelet therapy with clopidogrel or anticoagulation with coumadine was discontinued 7 days prior to cardiac surgery. In case of drug eluting stent implantation prior to the planned procedure, if possible surgery was defered for more than 4 weeks. Patients were eligible for inclusion in case of significant bleeding postoperatively after standard elective procedures. Patients with therapy-relevant known coagulation abnormalities (hemophilia-A /B, APC resistance, factor XIII deficiency, HIT-II etc.) were not included. In addition, patients that suffered live-threatening bleeding requiring immediate re-thoracotomy, mass-transfusion or ECMO support were excluded, as these cases usually are associated with multiple confounding variables that might not allow for a valid comparison of groups.

### Rotational 4-chamber Thromboelastometry (ROTEM®)

The ROTEM® (Pentapharm GmbH, Munich, Germany) system allows for „point-of-care “coagulation testing based on the classic thrombelastometry. Basic principles of the ROTEM® have been described in detail elsewhere [14]. Briefly, blood samples are activated and the time until first clot formation is measured (clotting time - CT). In addition maxium clot firmness (MCF) is assessed. The CT is mainly dependent on the availability of “intrinsic” and/or “extrinsic” coagulation factors and Heparin action, while the MCF depends on platelets function and fibrinogen. The ROTEM® system allows for four simultaneous measurements in separated chambers thus assessing the patient’s complete coagulation status with one run. Within the trial protocol the four chambers were utilized as follows: (1) INTEM: intrinsic pathway activation (2) HEPTEM: intrinsic activation, Heparin deactivated by Heparinase (3) FIBTEM: extrinsic activation, platelets inactivated (4) APTEM: extrinsic activation, added aprotinin. In addition, EXTEM (extrinsic pathway activation) was used in case of a reasonable suspicion for factor-VII deficiency.

### Intraoperative care

Surgery was performed according to clinical standards. In case serum calcium and pH were not within the standard range they were adjusted according to our internal standard operating procedures. During CPB aprotinin was administred with a loading dose of 1 to 2 Million IU followed by 0.5 Million IU per hour maintenance dose. Within the duration of the trial it has been withdrawn from the marked and was replaced by tranexamic acid. Tranexamic acid was administred with a loading dose of 10 mg/kg bodyweight over 20 min followed by a 1 mg/kg bodyweight/h infusion.

Special attention was applied to sufficient rewarming on cardiopulmonary bypass aiming at a body temperature (bladder) of 37 °C. After Heparin reversal, guided by repeat ACT measurements, coagulation products were administered in case of visible diffuse bleeding assessed by the surgeon on an empirical basis until standard coagulation test results were available. All patients received the same intraoperative care independent of their further course and potential randomization.

### Management in the ICU

After the procedure all patients were transferred to the intensive care unit. Extubation was considered as early as possible using a “fast-track” protocol. Arterial blood gas analyses were performed on a regular basis. Immediately after arrival at the ICU routine blood samples were sent to the laboratory including baseline coagulation testing (PTT, Quick, platelet count) in all patients according to routine practice. In case of significant bleeding (see definition above) patients were randomized to either ROTEM® or standard coagulation-test guided treatment using a computer-generated randomization list. The allocation sequence was concealed from the researcher (PMK) enrolling and assessing participants in sequentially numbered, opaque, sealed and stapled envelopes. A thin aluminium foil inside the envelope was used to render the envelope impermeable to intense light. Corresponding envelopes were opened only after the enrolled participants completed all baseline assessments and it was time to allocate the intervention (JK, EG). Randomization was performed immediately after arrival at the intensive care unit, because conventional laboratory test are usually not available on time in the operating room and furthermore comparison to a well-structured standard algorithm would then not have been possible. The allocation list was stored on a separate folder in the Coordinating Centre of Clinical Trials at the Heartcenter Leipzig.

For patients in the ROTEM®-group a four-chamber analysis was performed as described above. Further blood component therapy was performed according to the ROTEM®-guided protocol (Table 1). In patients randomized into the control group further “extended” standard coagulation test were sent to the laboratory and treatment was guided by an algorithm based on standard coagulation tests (Table 2).

**Table 1:** Transfusion protocol for the ROTEM®-guided group

Trigger:	drainage loss > 200 ml / h or 100 ml / 30 min at 30 min after arrival at the ICU
1.	4-chamber TEM (1 INTEM, 2 HEPTEM, 3 FIBTEM, 4 APTEM), Quick, AT3
2.	CT-INTEM/CT-HEPTEM> 1.5 = > **5000 IE Protamin**
3.	CT-HEPTEM> 260 s = > **FFP units** (kg body weight)^a^
4.	a: MCF-HEPTEM 35–45 mm and MCF-FIBTEM > 8 mm = > **1 platelet concentrate** b: MCF-HEPTEM< 35 mm = > **1 platelet concentrate**
5.	MCF-FIBTEM < 8 mm = > **2 g Fibrinogen**
6.	MCF-APTEM/MCF-HEPTEM > 1.5 or Aprotinin effective optically = > **2 Mio IE Aprotinin / 2 g Tranexamic acid**
	• In case of persistent bleeding - > re-testing according to (1) and further therapy as suggested by the protocol. • 2000 IE PPSB if INR > 2.0 and known liver dysfunction or previous Coumadin therapy. • 2000 IE AT3 if an increase > 50% (prothrombin time) is not to be expected via FFP-substitution.

Transfusion of RBCs according to the haemoglobin level of the blood-gas-analysis. Target value > 8.0 g/dl

^a^FFP (15 ml / kg KG): < 58 kg body weight - > 3 FFP, 58–75 kg body weight - > 4 FFP, 75–92 kg body weight - > 5 FFP, > 92 kg body weight - > 6 FFP

The entries in boldface represent the administered blood-components

**Table 2:** Transfusion protocol for the control group

Trigger:	drainage loss > 200 ml / h or 100 ml / 30 min at 30 min after arrival at the ICU
1.	Standard coagulation tests: platelets count, Fibrinogen, AT3, PTT, INR/Quick, α2-Antiplasmin, ACT
2.	ACT > 160 s = > **5000 IE Protamin (once)**
3.	PTT > 60 s = > **FFP units** (kg body weight)^a^ or if Quick < 50%
4.	Platelets count <100,103/μl = > **1 platelet concentrate**
5.	Fibrinogen <1,2 g/l = > **2 g Fibrinogen**
6.	α2-Antiplasmin <80% = > **2 Mio IE Aprotinin / 2 g Tranexamic acid**
	• In case of persistent bleeding - > re-testing according to (1) and further therapy as suggested by the protocol. • 2000 IE PPSB if INR > 2.0 and known liver dysfunction or previous Coumadin therapy. • 2000 IE AT3 if an increase > 50% (prothrombin time) is not to be expected via FFP-substitution.

Transfusion of RBCs according to the haemoglobin level of the blood-gas-analysis. Target value > 8.0 g/dl

^a^FFP (15 ml/kg KG): < 58 kg body weight - > 3 FFP, 58–75 kg body weight - > 4 FFP, 75–92 kg body weight - > 5 FFP, > 92 kg body weight - > 6 FFP

The entries in boldface represent the administered blood-components

The decision to perform a re-thoracotomy was made by the surgeon in charge of the patient independent of the respective group randomization.

Packed red blood cells (RBCs) were transfused triggered only by a haemoglobin level of less than 8 g/dl in the arterial blood-gas analysis. Preload (transfusion of crystalloid volume) was maintained liberally to avoid vasopressor therapy as far as possible. Care was taken to maintain sufficient body temperature by transfusing pre-warmed fluids and using forced-air warming blankets.

### Follow-up, data analysis and statistics

A power analysis was based on our initial experience with the ROTEM® analyzer and the results from previous studies using thrombelastography-guided coagulation management in cardiac surgery [11, 15]. The power analysis suggested that 30 patients per group would be required to demonstrate a 40% reduction in the use of allogeneic blood products with α of 0.05 and power of 80%.

Four patients were considered protocol violators, consequently 104 patients remained for the per-protocol analyses. Data were collected prospectively and 30-day follow-up was 100% complete. All statistical analyses were performed using SPSS, version 24.0 (Chicago, IL, USA). Continuous variables are expressed as mean ± standard deviation for Gaussian distributed variables and otherwise median values. For comparison of continuous variables the two-tailed Student’s t-test and, for non-normally distributed variables, the Mann-Whitney U-test were utilized. A *p*-value of less than 0.05 was considered statistically significant. The person performing data analysis and statistical calculations was blinded for the treatment modality. The study adheres to the current version of the CONSORT guidelines (Additional files 1 and 2) [16].

## Results

Between 03/2007 and 08/2009 104 patients met the inclusion criteria. Mean age was 67.2 ± 10.4 years, mean log EuroSCORE (European System for Cardiac Operative Risk Evaluation) was 7.0 ± 8.8%, and Society of Thoracic Surgeons (STS) Score was 2.4 ± 2.7%. Fifty patients (48%) received aortic valve replacement (AVR) and twenty-eight patients (26.9%) isolated CABG, whereas 25% received AVR in combination with isolated CABG. Mean CPB-time was 112.1 ± 55.1 min., mean cross-clamp time 72.5 ± 39.9 min., 13% of procedures were re-do operations and 4% required circulatory arrest. Baseline demographics were comparable in both groups. Detailed preoperative characteristics of the patients are given in Table 3. There was no major difference in the use of antiplatelet and/ or anticoagulation therapy prior to surgery except for a significantly higher rate of coumadin therapy within the ROTEM®-group (Table 3).

**Table 3:** Preoperative baseline characteristics of all 104 patients included in the study

Variables	OVERALL	CONTROL	ROTEM®	*p*-value
*Baseline values*				
Number, n (%)	104	52 (50)	52 (50)	
Age [years]	67.2 ± 10.4	68.1 ± 9.9	66.4 ± 12.9	0.62
Male sex, n (%)	84 (80.8)	45 (87)	39 (75)	0.21
BMI	26 [24.0;29.0]	25 [23.3;28.8]	27 [24.3;30.0]	0.11
Log. EuroSCORE [%]	7.0 ± 8.8	9.6 ± 9.8	8.2 ± 7.5	0.72
STS – Score [%]	2.4 ± 2.7	3.1 ± 3.1	3.3 ± 3.2	0.84
Biplane LVEF [%]	60.0 [45.0;65.0]	59.5 [45.0;65.0]	60.0 [50.0;65.0]	0.45
Diabetes mellitus, n (%)	33 (31.7)	16 (31)	17 (33)	1.00
COPD, n (%)	7 (6.7)	2 (4)	5 (10)	0.44
Creatinine [mmol/l]	91 [81.0;115.0]	92.5 [80.0;109.0]	101 [82.5;119.0]	0.11
Re-operation, n (%)	14 (13.5)	7 (14)	7 (14)	1.00
Deep hypothermic circulatory arrest, n (%)	4 (3.8)	2 (4)	2 (4)	1.00
Aortic valve surgery, n (%)	50 (48.0)	23 (44.2)	27 (51.9)	0.74
CABG, n (%)	28 (26.9)	16 (30.7)	12 (23.1)	0.41
Combined surgery^a^, n (%)	26 (25.0)	13 (25.0)	13 (25.0)	1.00
Acetylsalic acid, n (%)	62 (58)	33 (64)	29 (56)	0.54
Clopidogrel, n (%)	10 (9)	4 (8)	6 (12)	0.52
Coumadine, n (%)	8 (8)	1 (2)	7 (14)	0.03
LM Heparin, n (%)	21 (20)	9 (17)	12 (23)	0.47
Heparin, n (%)	10 (9)	3 (6)	7 (14)	0.20

*BMI* body mass index, *LVEF* left ventricular ejection fraction, *COPD* chronic obstructive pulmonary disease, *LM Heparin* low-molecular subcutaneous heparin. ^a^: Aortic valve surgery in combination with CABG. Data presented as numbers (%), mean ± SD or as median (interquartile range)

As shown in Table 4, perioperative values were not statistically different in both groups except for a higher heparin dose in the ROTEM®-group. Intraoperative transfusion requirements (prior to randomization) regarding red blood cells, platelets, plasma, fibrinogen, pooled factors or antifibrinolytic agents were comparable. At the time of the interim analysis, 75% more of the patients initially estimated were included in the study while the primary endpoint in the use of allogenic blood products could not be reached (Table 4).

**Table 4:** Clinical data of both randomized groups

Variables	CONTROL	ROTEM®	*p*-value
*Perioperative details*			
Number, n (%)	52 (50)	52 (50)	
CPB time [min.]	109.2 ± 74.6	119.8 ± 60.6	0.44
Cross-clamp time [min]	71.5 ± 51.0	77.3 ± 39.5	0.33
Heparine dosage [IU]	28,249.0 ± 10,419.0	33,364.6 ± 12,000.3	0.01
Protamine dosage [IU]	28,580.0 ± 9165.4	28,851.1 ± 8257.4	0.47
RBCs [units]	1.1 ± 1.7	1.0 ± 1.9	0.48
FFPs [units]	0.9 ± 1.7	0.5 ± 1.1	0.38
Platelet concentrates [units]	0.1 ± 0.5	0.2 ± 0.5	0.97
Fibrinogen [g]	0.1 ± 0.6	0.1 ± 0.4	0.71
PPSB [IU]	137.3 ± 566.4	135.4 ± 422.0	0.50
AT3 [IU]	137.3 ± 566.4	83.3 ± 331.6	0.72
Aprotinin, [Mio IU]	0.3 ± 0.7	1.1 ± 7.2	0.09
Tranexamic acid [g]	1.6 ± 1.5	1.9 ± 1.5	0.22
ACT (prior to ICU transfer) [sec]	135.4 ± 16.5	132.3 ± 20.3	0.09
Hct (prior to ICU transfer) [%]	28.9 ± 3.9	28.6 ± 4.1	0.70
Temperature (prior to ICU transfer) [°C]	36.3 [36.0;36.6]	36.4 [35.9;36.6]	0.78
*Postoperative - ICU*			
RBCs [units]	5.2 + 8.1	4.4 + 3.7	0.73
FFPs [units]	3.2 ± 4.7	2.2 ± 4.1	0.17
Platelet concentrates [units]	0.7 ± 1.1	0.4 ± 0.8	0.16
Fibrinogen [g]	0.2 ± 0.6	0.8 ± 1.1	0.01
PPSB [IU]	58.8 ± 420.1	81.6 ± 399.8	0.61
AT3 [IU]	19.6 ± 140.0	20.4 ± 142.9	1.00
Aprotinin [Mio IU]	0.5 ± 0.9	0.1 ± 0.4	0.01
Tranexamic acid [g]	0.1 ± 0.4	0.0 ± 0.3	1.00
Blood loss within first 24 h [ml]	1867.4 ± 827.4	1599.1 ± 834.3	0.07
Re-thoracotomy, n (%)	15 (29)	13 (25)	0.83
30-day mortality, n (%)	4 (8)	1 (2)	0.17
5-year mortality, n (%)	6 (12)	2 (4)	0.14

Data presented as numbers (%), mean ± SD or as median (interquartile range)

In the postoperative course (after randomization), the ROTEM®-based algorithm was associated with significantly less aprotinin, but increased fibrinogen usage. Requirements for RBCs, FFPs, platelets, PPSB, AT3 and tranexamic acid were comparable. Overall, there was a strong trend towards less bleeding within the first 24 h in the ROTEM® group visible however not reaching statistical significance (*p* = 0.066). Rate of re-thoracotomy for bleeding was comparable in both groups. A “surgical” bleeding could be identified in 21% (11 out of 15) in the control and in 17% (9 out of 13) in the ROTEM® group (*p* = 0.8).

Within 30 days, one patient in the ROTEM® (2%) and 3 patients in the control group (6%) suffered a stroke (*p* = 0.618). Requirement for dialysis due to acute renal failure was 6% (*n* = 3) in the control and 12% (*n* = 6) in the ROTEM® group (*p* = 0.488). Ventilation times were prolonged but comparable in both groups (control: 95.9 ± 197.2 vs. ROTEM®: 97.7 ± 159.3 h; *p* = 0.17).

Thirty-day mortality rate was 8% (*n* = 4) in the control and 2% (*n* = 1) in the ROTEM® group (*p* = 0.17). The patient in the ROTEM® group died on postoperative day (POD) 12 due to multi-organ failure. In the control group, one patient died due to acute myocardial infarction (POD 3), one due to right heart failure (POD 29), one due to unclear reasons (POD 15) and one due to multi-organ failure (POD 27). At 5-year follow-up mortality rate was 12% (*n* = 6) in the control and 4% (*n* = 2) in the ROTEM® group (*p* = 0.14).

### Patients at “high-risk” for bleeding (long CPB times)

Out of the total 104 patients 55 patients were identified that underwent procedures with a long CPB-time known to increase the risk for diffuse coagulopathy. As shown in Table 5, baseline and perioperative values were not statistically different in both treatment groups and intraoperative transfusion requirements again were comparable although ROTEM® patients received more aprotinin intraoperatively.

**Table 5:** Clinical characteristics of 55 patients with “long” CPB time (≥ 115 min)

Variables	CONTROL	ROTEM®	*p*-value
*Baseline values*			
Number, n (%)	26 (47)	29 (53)	
Age [years]	66.9 ± 11.1	66.7 ± 10.0	0.87
Male sex, n (%)	22 (85)	26 (90)	0.70
BMI	24.5 [23.0;29.0]	27.0 [25.0;29.5]	0.07
Log. EuroSCORE [%]	10.2 ± 10.5	7.4 ± 6.9	0.40
STS – Score [%]	3.7 ± 3.9	3.3 ± 3.1	0.73
Biplane LVEF [%]	60.0 [41.5;65.0]	59.0 [50.0;65.0]	1.00
Diabetes mellitus, n (%)	7 (27)	7 (24)	1.00
COPD, n (%)	1 (4)	5 (17)	0.20
Creatinine [mmol/l]	98.0 [80.0;109.3]	106.0 [86.5;130.5]	0.07
Deep hypothermic circulatory arrest, n (%)	2 (8)	1 (3)	0.60
Re-operation, n (%)	2 (8)	4 (14)	0.67
*Perioperative details*			
CPB time [min]	161.4 ± 59.8	159.3 ± 41.3	0.93
Cross-clamp time [min]	105.8 ± 41.9	99.3 ± 31.7	0.84
Heparin [IU]	31,528.0 ± 9526.2	34,925.9 ± 10,049.6	0.16
Protamin [IU]	30,720.0 ± 7924.2	31,000.0 ± 5699.1	0.46
RBCs [units]	1.4 ± 2.0	1.2 ± 2.1	0.45
FFPs [units]	1.2 ± 1.9	0.7 ± 1.2	0.52
Platelet concentrates [units]	0.3 ± 0.7	0.1 ± 0.4	0.20
Fibrinogen [g]	0.2 ± 0.7	0.1 ± 0.5	1.00
PPSB [IU]	200.0 ± 707.1	166.7 ± 500.0	1.00
AT3, IU, mean ± SD	200.0 ± 707.1	129.6 ± 429.5	1.00
Aprotinin [Mio IU]	0.6 ± 0.9	1.9 ± 9.6	0.03
Tranexamic acid [g]	1.6 ± 1.5	1.8 ± 1.5	1.00
ACT (prior to ICU transfer) [sec]	134.1 ± 16.1	130.7 ± 20.2	0.18
Hct (prior to ICU transfer) [%]	28.1 ± 4.4	28.4 ± 3.8	0.71
Temperature (prior to ICU transfer) [°C]	36.4 [36.0;36.6]	36.3 [36.0;36.6]	0.70
*Postoperative - ICU*			
RBCs [units]	7.1 + 10.8	3.8 + 3.8	0.28
FFPs [units]	4.2 ± 5.6	1.7 ± 2.8	0.07
Platelet concentrates [units]	1.1 ± 1.3	0.4 ± 0.6	0.02
Fibrinogen [g]	0.2 ± 0.6	0.9 ± 1.2	0.01
PPSB [IU]	0.0 ± 0.0	0.0 ± 0.0	1.00
AT3 [IU]	40.0 ± 200.0	0.0 ± 0.0	0.48
Aprotinin [Mio IU]	0.4 ± 0.8	0.0 ± 0.0	0.02
Tranexamic acid [g]	0.1 ± 0.4	0.1 ± 0.4	1.00
Blood loss within first 24 h [ml]	2056.8 ± 974.5	1538.2 ± 806.4	0.03
Re-thoracotomy, n (%)	7 (27)	6 (21)	0.75
30-day mortality, n (%)	3 (12)	0 (0)	0.10
5-year mortality, n (%)	4 (15)	0 (0)	0.03

Data presented as numbers (%), mean ± SD or as median (interquartile range)

**Fig. 1:**
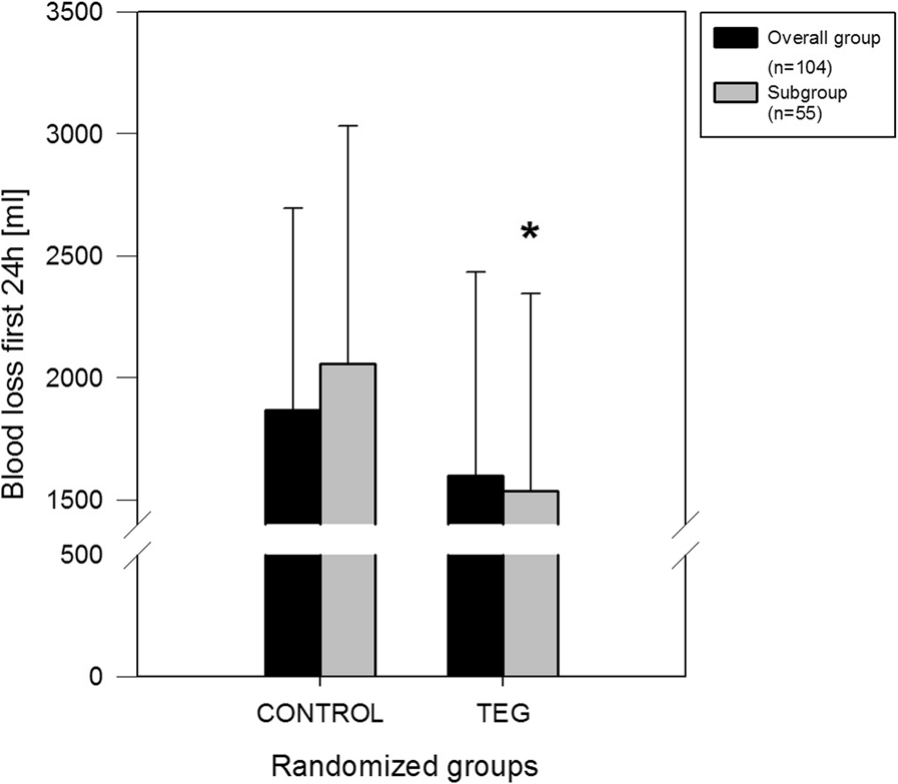
* evidence for a significant difference between control group and ROTEM®-guided therapy in the subgroup of patients with long CPB-times (≥ 115 min.; p = 0.032)

After randomization (postoperative course), the two different treatment protocols resulted in significantly different distribution of coagulation products requirements. Whereas ROTEM® patients received significantly less platelets concentrates and aprotinin, control patients required less fibrinogen. Re-thoracotomy rate was comparable and 3 patients in the control versus none in the ROTEM® group died. At 5-year follow-up there was a significant improved survival in the ROTEM® group (0% vs. 15%; *p* = 0.03). Overall, ROTEM® patients suffered significantly less blood loss within the first 24 h (ROTEM®: 1538.2 ± 806.4 ml vs. control: 2056.8 ± 974.5 ml; *p* = 0.032; Fig. 1).

### Cost analysis

**Fig. 2:**
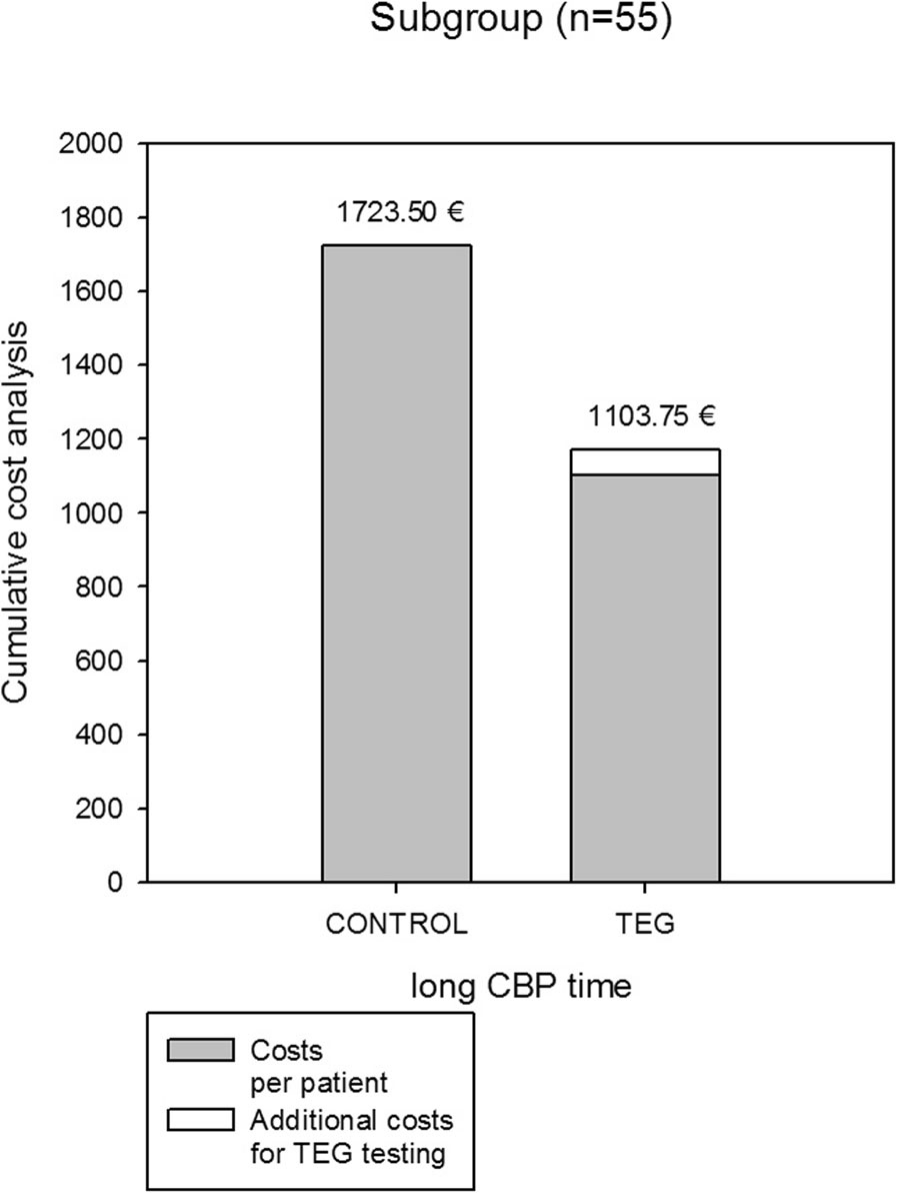
A cumulative cost analysis of all blood component products in the subgroup of patients with “long” CPB-times revealed cost savings of 619.8 € per patient treated in the “point-of-care” 4-chamber ROTEM®. Costs savings were not annihilated by the additional costs for ROTEM® testing (on average: 68.3 € per patient)

A cumulative cost analysis of all blood component products revealed cost savings of 128.50 € per patient (− 10%) treated in the “point-of-care” 4-chamber ROTEM® group (Table 6). However, in the subgroup of patients with “long” CPB-times cost savings increased to even 619.75 € per patient (Table 7, Fig. 2). In total, average costs of all blood component products per patient decreased in this group from 1723.50 € to 1103.75 € (− 36%). In addition, costs savings were not annihilated by the additional cost for ROTEM® testing (on average: 68.30 € per patient).

**Table 6:** Cumulative cost analysis of all blood products (*n* = 104 patients)

Variables	CONTROL	ROTEM®
Number	52 (50)	52 (50)
*intraoperative*		
RBCs (70 €)	3780	3290
FFPs (51 €)	2346	1326
Platelet concentrates (unit: 500 €)	4000	3500
Fibrinogen, 1 g (287.5 €)	2013	1725
PPSB, 500 IU (120 €)	1680	1560
AT3, 1000 IU (70 €)	490	280
Aprotinin (2.5 Mio IU 123.75 €)	668	2574
Tranexamic acid (500 mg: 7.63 €)	1206	1373
Cumulative costs	16,183	15,628
*ICU (after randomization)*		
RBCs (70 €)	18,690	14,980
FFPs (51 €)	8313	5559
Platelet concentrates (unit: 500 €)	17,500	10,000
Fibrinogen, 1 g (287.5 €)	2875	11,500
PPSB, 500 IU (120 €)	720	960
AT3, 1000 IU (70 €)	70	70
Aprotinin (2.5 Mio IU 123.75 €)	1188	198
Tranexamic acid (500 mg: 7.63 €)	61	31
Cumulative costs	49,417	43,298
Overall cumulative costs [€]	65,600	58,926
Costs per patient [€]	1261.5	1133
Costs savings per patient [€]	–	128.5

**Table 7:** Cumulative cost analysis of all blood products (*n* = 55 patients with CPB time ≥ 115 min)

Variables	CONTROL	ROTEM®
Number	26 (47%)	29 (53%)
*intraoperative*		
RBCs (unit: 70 €)	2520	2240
FFPs (unit: 51 €)	1530	1020
Platelet concentrates (unit: 500 €)	4000	1500
Fibrinogen (1 g: 287.5 €)	1438	1150
PPSB (500 IU: 120 €)	1200	1080
AT3 (1000 IU: 70 €)	350	245
Aprotinin (2.5 Mio IU 123.75 €)	668	2574
Tranexamic acid (500 mg: 7.63 €)	595	732
Cumulative costs [€]	12,301	10,541
*ICU (after randomization)*		
RBCs (unit: 70 €)	12,460	7140
FFPs (unit: 51 €)	5304	2397
Platelet concentrates (unit: 500 €)	13,000	5000
Fibrinogen (1 g: 287.5 €)	1150	6900
PPSB (500 IU: 120 €)	0	0
AT3 (1000 IU: 70 €)	70	0
Aprotinin (2.5 Mio IU: 123.75 €)	495	0
Tranexamic acid (500 mg: 7.63 €)	31	31
Cumulative costs [€]	32,510	21,468
Overall cumulative costs [€]	44,811	32,009
Costs per patient [€]	1723.5	1103.8
Costs savings per patient [€]	–	619.8

## Discussion

Significant postoperative bleeding is a problem well known in any cardiac surgical center. It has to be expected in 5–10% of patients depending on the type of surgery and potential pre-existing risk factors [3]. It has been demonstrated that transfusion requirements have a considerable impact on postoperative outcome and are associated with increased mortality [17].

Optimally, treatment algorithms would be based on coagulation tests with rapid results, independent of heparin and capable of assessing the current function of the different coagulation system components. Theoretically, modified thrombelastometry systems designed as a “point-of-care” test seems well suited and have been proven to further reduce transfusion requirements in a small prospectively randomized trial compared to an algorithm solely based on standard tests [18].

An advanced “point-of-care” system (ROTEM®) has been introduced that allows for simultaneous testing in four chambers with different activation agents (“intrinsic” / “extrinsic”), platelets blockade and aprotinin or heparinase addition. The clinical introduction of this thrombelastometry-based blood component treatment has been demonstrated to reduce transfusion requirements and costs [11, 15, 19, 20]. The aim was to study a thrombelastometry-based algorithm in comparison to a protocol based on classic coagulation tests known to be superior to “empiric” treatment.

Overall we observed comparable transfusion requirements between the ROTEM®-guided and the classic coagulation test guided group regarding RBCs, FFPs, platelets and pooled factors (PPSB). Our ROTEM®-guided blood component treatment algorithm was at least as effective and safe as protocols based on classic coagulation tests. However, distribution blood products differed between both groups. ROTEM® patients received more fibrinogen but significantly less aprotinin, as already stated in a recent study [11].

There was a clear trend towards less 24 h-bleeding visible in the ROTEM® group, also more patients were on coumadin treatment preoperatively and heparin dosages were significantly higher in the ROTEM® group. When analyzing the subgroup of “high-risk” patients with long CPB-times known to be prone to diffuse coagulopathy ROTEM® guided treatment resulted in significantly less 24 h-drainage loss and an improved 5-year survival. It seems that the more specific approach of the ROTEM® based algorithm resulted in less bleeding. In addition, the ability to deliver the specific treatment faster than with standard tests may have contributed to the observed benefit. The ROTEM® protocol requires 10 min until the results allow for a decision if additional protamine, FFPs or platelets are required and after 30 min measurements will guide platelets, fibrinogen or antifibrinolytic agent therapy. In contrast, at least in our center, results of standard coagulation test are rarely available in less than one hour.

Rate of re-thoracotomy was not different between the two groups. To differentiate between surgical bleeding and diffuse coagulopathy is always a difficult task. Standard coagulation tests have been shown to be of no help at all in this scenario: negative predictive accuracy has been reported with 50% - basically the same as guessing [5]. In contrast, ROTEM® is more accurate and allows for a negative predictive value (excluding diffuse coagulopathy) of 82%, however positive prediction is less [5, 21]. In conclusion, ROTEM® might be helpful in the assessment of surgical versus diffuse bleeding but overall accuracy seems to be not sufficient. Thus, the decision for re-thoracotomy in our series was based on clinical judgment and only partially based on ROTEM® or standard coagulation test. As in only 21% of control and in 17% of ROTEM® patients a surgical bleeding could be identified, a substantial number of bleeding events could have been avoided with a better decision making protocol. Ideally, specific predictive values derived from ROTEM® or other coagulation tests might become available in the future facilitating a more evidence based approach if or if not to perform a re-thoracotomy [22, 23].

Avarage costs for the blood products used in this study were estimated according to Spalding et al. [11]. Consistent with previous studies we could confirm in our prospective randomized trial a cost-reduction when using a specific ROTEM® based treatment algorithm (Tables 6 and 7). Cost-savings were not counterbalanced by the additional costs for thrombelastometry testing (Fig. 2). Thus, it has to be considered that the implementation of such a ROTEM®-based protocol requires substantial human training and a dedicated and highly motivated ICU team as the work-load is increased due to the requirements to manually perform the ROTEM® tests on the ICU in comparison to simply sending a blood sample to the laboratory for traditional coagulation tests.

Another issue of concern is the accuracy and reproducibility of a “point-of-care” test performed manually by “non-specialized” physicians outside the laboratory environment. However, for the ROTEM® device sufficient reproducibility and stability of measurements have been reported [24–26]. The use of fully-automated systems like ROTEM® sigma need to be disseminated more widely to stimulate a broader use of this beneficial technology.

We observed a beneficial effect of the ROTEM® algorithm predominantly in the subgroup of “high-risk” patients with prolonged CPB-times. However, we have to consider that due to the study design ROTEM® was compared to a well-structured and evidence based standard transfusion protocol.

In this study, there was an overall trend towards improved survival at 30-days, however in the subgroup of “high-risk” patients known to be prone to diffuse coagulopathy a significant reduction in 5-year mortality was proven in the long-term follow-up. Standard treatment algorithms have been proven to significantly reduce transfusion requirements in comparison with “empiric” therapy [1, 5, 7, 27]. Hence, the observed benefit of a ROTEM®-guided protocol has been shown to be effective and safe in a prospective randomized trial in cardiac surgical patients that suffer postoperative bleeding and secondly a further reduction of bleeding, costs and mortality could be demonstrated. In our opinion, “empiric” blood component therapy should not be used in clinical practice. For regular patients both, standard and ROTEM®-based specific treatment algorithms have been shown repeatedly superior providing a true clinical benefit for the patients [1, 5].

With regard to a recent updated meta-analysis by Serraino and collegues [28] ROTEM-guided algorithms lead to a significant reduction in transfusion of RBCs, FFPs, platelets and the rate of severe acute kidney injury compared to CONTROL groups. There was also an improvement seen in mortality, number of reoperations for bleeding, ventilation times, shorter ICU length of stay and hospital stay, but none of them significantly reduced. The authors concluded that viscoelastic testing lacks clinical effectiveness with only weak evidence and low predictive accuracy for coagulopathic bleeding. However, a treatment algorithm based on “point-of-care” 4-chamber ROTEM® seemed to be at least as effective as standard therapy with improvement in a broad range of relevant clinical parameters. Furthermore, our incidence of postoperative acute kidney injury was rather low for a sample of patients with significant bleeding. Interestingly, our results are supported by newer studies, who demonstrated that a low nadir hematocrit (cutoff value of about 24%) was inversely associated with acute kidney injury [29–31].

A ROTEM® guided protocol seems to be capable to further reduce bleeding, costs and mortality as it allows for a highly specific and fast therapy tailored to the functional coagulation status of the individual patient and might be especially beneficial in “high-risk” patients prone to bleeding complications [14].

### Limitations

The major limitation of this trial is that patients with aortic valve replacement as well as with combined CABG were included. Thus, our data might include a considerable preoperative inhomogeneity regarding the incidence of von-Willebrand-Syndrom. In addition, limited evidence is available in regard to the structure of the ROTEM® algorithm and baseline reference values [32] which might not allow comparability between “regular” and cardiac surgical patients after CPB. Furthermore, the small number of patients as well as surgical bleeding, that cannot be treated based on thromboelastometry, may have biased our results. However, it is still a unique randomized trial assessing ROTEM® effectiveness in cardiac surgical patients in case of postoperative bleeding. Furthermore, the rate of inadequate platelet response was not assessed in our study, but might be equally distributed within the groups due to randomization.

Within the duration of the trial Trasylol had been withdrawn from the marked. Subsequently, aprotinin was replaced by tranexamic acid. However, this change in treatment affected both groups equally and in addition tranexamic acid has been shown to be as effective as aprotinin in several trials [33].

## Conclusion

Significant postoperative bleeding after cardiac surgical procedures is a well-known problem with dramatic impact on clinical outcome, mortality and costs. Based on the results of our randomized trial comparing a “4-chamber” modified thromboelastometry-guided protocol with an algorithm based on standard coagulation testing the ROTEM® approach proved to be at least as effective and safe as the standard approach. In patients with long CPB-times prone for diffuse coagulopathy ROTEM®-guided therapy may result in less bleeding, a marked reduction in costs and long-term mortality.

## Supplementary information

**Additional file 1.** CONSORT 2010 Checklist, CONSORT 2010 checklist of information to include when reporting a randomised trial.

**Additional file 2.** CONSORT 2010 Flow Diagram, Flow diagram of the progress through the phases of the prospective randomised trial of the ROTEM® and CONTROL group.

## Data Availability

The trial protocol, datasets used and/or analysed during the current study are available from the corresponding author on reasonable request.

## Abbreviations

ACT: activated clotting time (last value in the operating theatre)
APC: activated protein C
APTEM: rotational thromboelastometry that shows hyperfibrinolysis by inhibition with aprotinin
AT3: anti-thrombin 3
AVR: aortic valve replacement
CABG: coronary artery bypass graft
CONSORT: Consolidated Standards of Reporting Trials
COPD: chronic obstructive pulmonary disease
CPB: cardiopulmonary bypass
CT: clotting time
dl: deziliter
EuroSCORE: European System for Cardiac Operative Risk Evaluation
FFPs: fresh frozen plasma
FIBTEM: rotational thromboelastometry that shows isolated fibrinogen contribution
Hct: hematocrit
HEPTEM: rotational thromboelastometry that shows heparin effects by inhibition with heparinase
HIT: heparine induced thrombocytopenia
ICU: intensive care unit
INR: international normalized ratio
INTEM: rotational thromboelastometry that shows intrinsic thromboelastometry
IU: international units
kg: kilogramm
LVEF: left ventricular ejection fraction
MCF: maxium clot firmness
min: minute
ml: mililiter
mm: milimeter
N: number
POD: postoperative day
PPSB: prothrombin complex concentrate: pooled factors (Prothrombin-Proconvertin-Stuart Factor-Antihemophilic Factor B)
PTT: partial thromboplastin time
RBCs: packed red blood cells
ROTEM: rotational thromboelastometry
Sec: second
STS: Society of Thoracic Surgeons Score
