# Supplementary material for: Thrombelastometry guided blood-component therapy after cardiac surgery: a randomized study
Source: BMC Anesthesiol. 2019 Nov 6;19:201. doi: 10.1186/s12871-019-0875-7 (PMC6833285; doi:10.1186/s12871-019-0875-7)
Supplement: Supplementary file 2 — Additional file 2. CONSORT 2010 Flow Diagram, Flow diagram of the progress through the phases of the prospective randomised trial of the ROTEM® and CONTROL group. [file 12871_2019_875_MOESM2_ESM.doc]

**
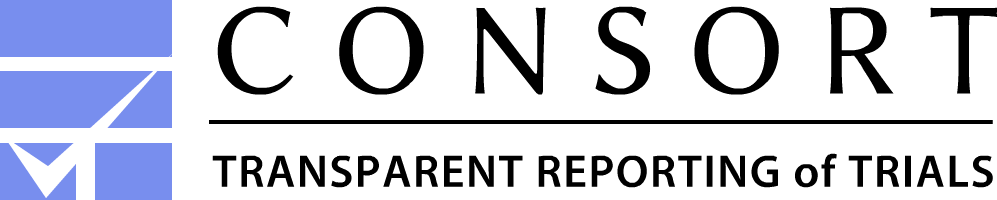
**

**CONSORT 2010 Flow Diagram**

**Allocation**

**Analysis**

**Follow-Up**

**Enrollment**

Assessed for eligibility (n = 108)

Excluded (n = 4)

♦  Not meeting inclusion criteria (n = 3)

♦  Declined to participate (n = 1)

♦  Other reasons (n = 0)

Analysed (n = 52)
♦ Excluded from analysis (give reasons) (n= 0)

Lost to follow-up (give reasons) (n = 0)

Discontinued intervention (give reasons) (n= 0)

Allocated to CONTROL (n = 52)

♦ Received allocated intervention (n = 52)

♦ Did not receive allocated intervention (give reasons) (n = 52)

Lost to follow-up (give reasons) (n = 0 )

Discontinued intervention (give reasons) (n= 0)

Allocated to ROTEM® (n = 52)

♦ Received allocated intervention (n = 52)

♦ Did not receive allocated intervention (give reasons) (n = 52)

Analysed (n = 52)
♦ Excluded from analysis (give reasons) (n= 0)

Randomized (n = 104)
